# How WRKY transcription factors fine-tune specificity in plant stress responses: from W-box to regulatory code

**DOI:** 10.1007/s11033-026-12354-0

**Published:** 2026-07-10

**Authors:** Srushtideep Angidi, Khizar Razzaq

**Affiliations:** Department of Plant Pathology, North Dakota State University, Fargo, ND 58102 USA

**Keywords:** WRKY transcription factors, Regulatory code, Chromatin accessibility, Multi-omics, Artificial intelligence

## Abstract

WRKY transcription factors are among the largest plant-specific transcription factor families and play central roles in coordinating gene expression during biotic and abiotic stress. Despite decades of research, a fundamental paradox remains: WRKY proteins bind short, widely distributed W-box cis-elements yet generate highly selective, context-dependent transcriptional outputs in vivo. In this review, we argue that WRKY specificity is not determined by DNA binding alone but emerges from the integration of five regulatory layers: cis-regulatory motif grammar, chromatin accessibility and epigenetic state, protein–protein interactions, post-translational modifications and proteostasis, and signaling context, which we collectively define as the WRKY regulatory code. We discuss how chromatin state controls which genomic W-box sites are physically accessible, while post-translational modifications and cofactor interactions determine which accessible sites are productively engaged and whether the transcriptional output is activation or repression. Representative mechanistic examples illustrate how different combinations of these regulatory layers generate precise, condition-dependent transcriptional programs. We further discuss how multi-omics integration and machine learning-based predictive modeling can decode and empirically test this regulatory code across stresses, tissues, and developmental contexts. The regulatory code perspective resolves key inconsistencies in WRKY biology and provides a practical conceptual framework for more targeted manipulation of stress-responsive transcriptional networks in crops.

## Introduction

WRKY transcription factors (TFs) are among the largest plant-specific transcription factor families and have been studied extensively for two decades. After initial identification of WRKY transcription factors as regulators of pathogen-responsive gene expression, studies on WRKY proteins have expanded rapidly across a wide range of plant species and stress conditions. Despite detailed analyses of WRKY gene expression profiles, loss and gain of function phenotypes, and stress associated regulatory roles, the molecular mechanisms that confer target gene specificity to WRKY transcription factors remain unclear [11, 20, 37, 46].

The defining structural feature of the WRKY family, including the highly conserved WRKY DNA-binding domain and its interaction with W-box cis-elements (TTGACC/T) in the promoters of target genes, was studied in early research. These discoveries positioned WRKY TFs as central regulators of stress responses, particularly in plant immunity and pathogen defense [16, 20]. Genome-wide studies and functional studies have expanded the catalog of WRKY genes across plant species and linked individual WRKYs to a wide range of physiological processes, including secondary metabolism, hormone signaling, senescence, and tolerance to drought, heat, cold, salinity, and pathogen attack [11, 35, 37, 38]. As the research progressed in this area, individual WRKY transcription factors were frequently found to control extensive and partially overlapping gene networks, often functioning across multiple stress responses and signaling pathways. In parallel, the regulatory role of a given WRKY protein was shown to be highly context dependent, acting as a transcriptional activator or repressor depending on factors including the developmental stage, and intensity or duration of the stress stimulus. Functional versatility for numerous WRKY members has been documented in both model plant systems and agronomically important crops, highlighting the dynamic and context-specific nature of WRKY-mediated transcriptional regulation [3, 35]. The relationship between WRKY transcription factors and their downstream gene targets raises a fundamental and still unresolved question: by what molecular mechanisms WRKY proteins select specific target genes and generate distinct transcriptional outputs under various stress conditions [21]. A major limitation is the reliance on expression-based functional analyses to infer WRKY function. Most of the studies define biological roles primarily based on stress-induced transcriptional activation or repression, supported by overexpression or loss of function phenotypes [1, 44], which establish WRKY transcription factors as key regulators of abiotic and biotic stress responses, providing the limited mechanistic insight into how regulatory specificity is achieved at the molecular level [3]. Together, these findings suggested that WRKY transcriptional specificity cannot be explained by DNA binding alone, but depends on additional regulatory layers, including post-translational modifications, interactions with other proteins, epigenetic context, chromatin accessibility, and coordination with parallel transcriptional regulators, all of which are essential for modulating WRKY target gene selection and transcriptional responses under stress conditions [9, 26]. The widespread distribution of W-box cis-elements across plant genomes complicates functional interpretation of WRKY transcription factors. While thousands of genes harbor degenerate W-box motifs within their promoter regions, only a limited fraction of these loci are transcriptionally regulated by WRKYs in each cellular context. Promoter occupancy analysis and genome-wide assays have consistently demonstrated that the mere presence or frequency of W-box elements is a poor predictor of WRKY binding and transcriptional regulation, which highlights the limitation of motif-centric models of gene control [4, 15]. Recent in vivo and chromatin-based studies further support that W-box recognition is necessary but not sufficient for WRKY target specificity, indicating that WRKY binding and regulatory activity are constrained by additional layers of regulation [3, 6, 40]. Recent comprehensive reviews of the WRKY research field have highlighted the growing complexity of WRKY-driven transcriptional networks and emphasized that WRKY function is shaped not by DNA binding but by chromatin dynamics, protein-protein interactions, and integration with multiple signaling pathways [28, 35, 46]. Despite the abundance of WRKY studies, the field still lacks a coherent framework that explains how WRKY transcription factors generate precise, condition-dependent transcriptional outputs from broadly distributed cis-elements.

In this review, we argue that WRKY specificity should be understood as an emergent property of a multi-layered regulatory code, rather than as a direct consequence of W-box binding alone. Here, we use the term “WRKY regulatory code” to describe the combinatorial integration of five regulatory layers: (i) cis-regulatory motif grammar, (ii) chromatin accessibility and epigenetic state, (iii) protein–protein interaction networks, (iv) post-translational modifications and proteostasis, and (v) signaling context across stress, tissue, and time. Together, these layers determine whether a WRKY transcription factor can access a potential target locus, whether it is transcriptionally competent, which cofactors it recruits, and whether the final output is activation, repression, or no measurable response. By shifting the focus from individual WRKY genes to the mechanisms that govern target selection and transcriptional outcome, this review provides a conceptual framework for explaining how WRKY transcription factors generate precise, condition-dependent stress responses. Ultimately, adopting a regulatory-code perspective may help resolve longstanding ambiguities in WRKY biology and provide a foundation for more predictive manipulation of stress-responsive transcriptional networks in crops.

## Cis-regulatory logic of W-box recognition

WRKY transcription factors are defined by their capacity to recognize and bind W-box cis-regulatory elements, characterized by the consensus sequence TTGACC/T, within the promoters of target genes [47]. This interaction is mediated by a highly conserved WRKY DNA-binding domain and constitutes the core molecular mechanism underlying WRKY-dependent transcriptional regulation. Genetic and functional studies have established that W-box binding is essential for WRKY activity, positioning this motif as a central regulatory element in stress-responsive gene expression, particularly in plant immune and defense pathways [2, 20, 37].

Structural and mutational analyses have provided mechanistic insight into W-box recognition, demonstrating that the conserved WRKYGQK motif directly contacts the core nucleotides of the W-box cis-element and confers sequence-specific DNA binding by WRKY transcription factors [19, 50]. Consistent with these findings, biochemical binding assays revealed that mutations within the W-box core sequence substantially reduce WRKY-DNA binding affinity, supporting the view that canonical W-box motifs represent preferred docking sites for WRKY proteins [4]. Importantly, not all W-box motifs are functionally equivalent: flanking nucleotide context, motif density and clustering, the relative spacing of adjacent W-boxes, and the presence of co-occurring regulatory elements collectively modulate WRKY binding affinity and transcriptional output in a promoter-specific manner [4, 15]. This cis-regulatory grammar means that W-box elements function as permissive sites defining potential WRKY responsiveness, rather than as deterministic switches guaranteeing activation or repression.

Despite their importance for WRKY binding, canonical W-box motifs are highly abundant across plant genomes and occur in the promoters of thousands of genes irrespective of stress responsiveness or transcriptional regulation [40]. This widespread distribution raises a fundamental conceptual problem: if WRKY transcription factors recognize a short and prevalent cis-element, how is selective and context-dependent gene regulation achieved? Importantly, accumulating evidence indicates that WRKY-W-box interactions are not binary but quantitative in nature, with binding affinity and regulatory output varying substantially across different promoter contexts and physiological conditions. In this framework, W-box elements function as permissive cis-regulatory sites that define potential WRKY responsiveness rather than deterministic switches that guarantee transcriptional activation or repression. The disconnect between pervasive W-box occurrence and the selective nature of WRKY-dependent transcription suggests that core motif recognition alone is insufficient to confer regulatory specificity. Instead, WRKY target selection and transcriptional outcomes are formed by additional layers of regulation that operate in combination with W-box binding, including cis-element variation, protein-protein interactions, chromatin accessibility, and signaling-dependent modulation of WRKY activity [1], which provide a conceptual foundation for exploring multiple regulatory dimensions that integrate with W-box recognition to fine-tune WRKY-mediated transcription under stress conditions.

## Chromatin and epigenetic gating of WRKY occupancy

In vivo, WRKY binding is constrained by chromatin, where DNA methylation, nucleosomes, and histone marks determine whether W-boxes are physically accessible and binding is coupled to transcriptional output [43]. Plant stress epigenetics emphasizes that regulatory DNA is dynamically gated during stress through coordinated changes in histone modifications, methylation, and chromatin remodeling, shaping the transcription factor access at a genome-wide scale [7].

Methylation-dependent exclusion is a direct mechanism relevant to WRKY specificity. Plant cytosine methylation can manipulate TF binding through chromatin compaction and by interfering with TF-DNA contacts [22]. Single-cytosine methylation within a W-box can lower the binding by multiple WRKY domains, demonstrating that an optimal motif becomes nonfunctional when epigenetically masked [8]. Histone-linked regulation provides a second route to gating, for WRKYs, which can overlap with chromatin-modifying machinery. For example, WRKY53 was found to be involved in complexes with histone deacetylation components, demonstrating how WRKY activity can be manipulated by chromatin-associated cofactors rather than DNA binding alone [10]. Beyond this, stress-induced chromatin remodeling can create time-limited accessibility windows. For example, heat-stress studies showed coordinated roles for histone deacetylase activity and remodeling complexes in transcriptional programs, providing a mechanistic guide for condition-dependent exposure of cis-elements [5].

Direct in vivo evidence further links chromatin context to WRKY occupancy during immunity. The receptor-like kinase ERECTA (ER) promotes Arabidopsis resistance to *Sclerotinia sclerotiorum* through the SWR1 chromatin remodeling complex. Cai et al., [6] showed that ER signaling and SWR1 are required for enrichment of the H2A.Z histone variant and the active H3K4me3 mark at promoters of WRKY33 target genes, thereby facilitating WRKY33 binding upon pathogen challenge. This study provides a mechanistic demonstration that promoter chromatin state can actively shape WRKY target selection during immune responses. Complementary genome-wide ChIP-seq analysis of WRKY18, WRKY40, and WRKY33 after flg22 elicitation revealed that each factor occupies more than 1,000 loci in vivo, with binding largely induced by immune activation despite the broad genomic distribution of W-box motifs [3]. These findings reinforce that WRKY occupancy is highly context dependent and cannot be inferred from motif presence alone.

**Fig. 1:**
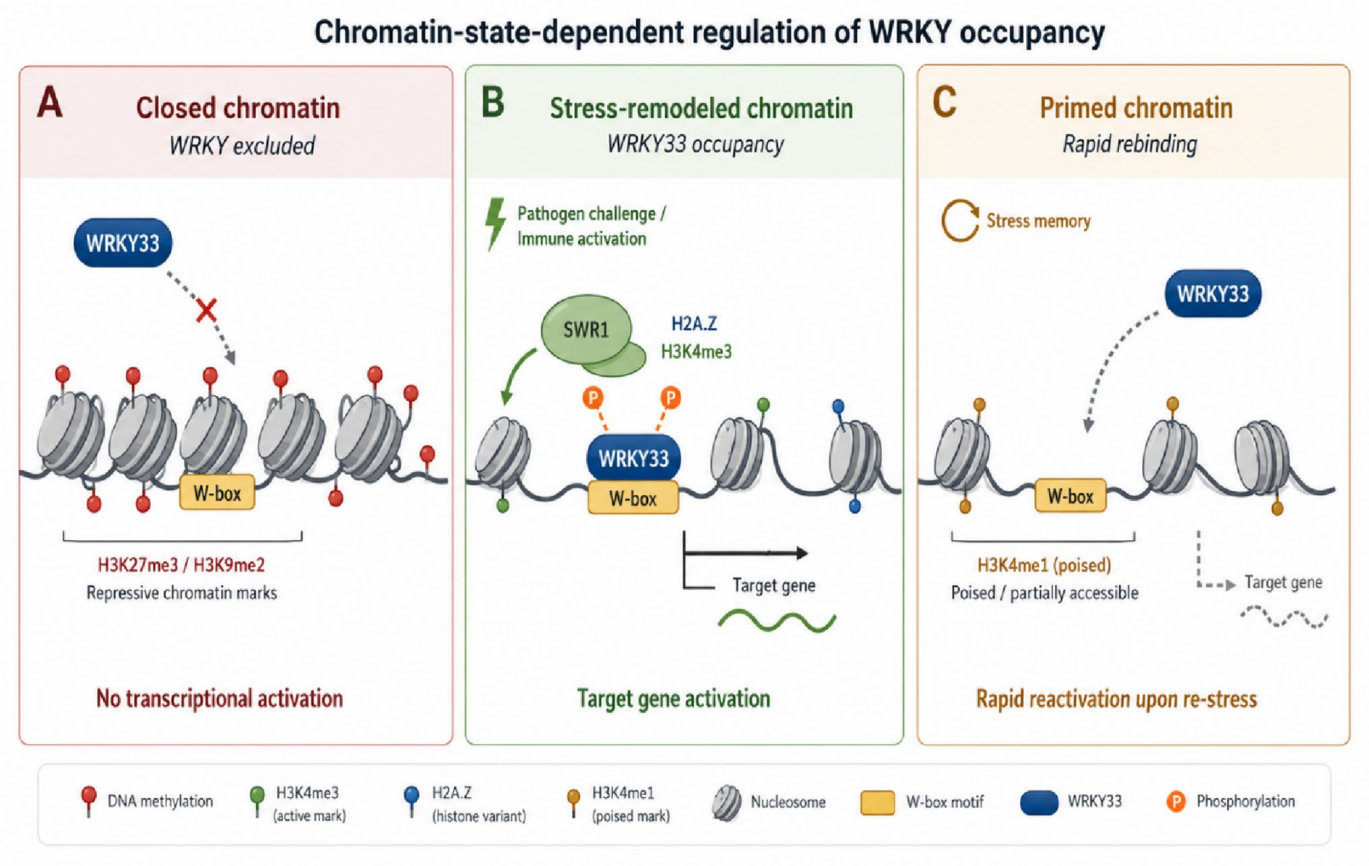
Chromatin-state-dependent regulation of WRKY transcription factor occupancy. WRKY target selection is constrained by promoter chromatin state. (**A**) In closed or methylated chromatin, dense nucleosome occupancy, DNA methylation, and repressive histone marks such as H3K27me3 and H3K9me2 restrict access to W-box cis-elements, preventing WRKY binding and transcriptional activation. (**B**) During immune activation, stress-induced chromatin remodeling exposes W-boxes at promoters. In the WRKY33-centered model, SWR1-associated remodeling, H2A.Z deposition, and enrichment of the active H3K4me3 mark promote an accessible chromatin state that enables WRKY33 occupancy and activation of defense-related target genes. Post-translational regulation, including phosphorylation, can further refine transcriptional output. (**C**) Following prior stress exposure, a primed or poised chromatin configuration may retain partial accessibility at responsive promoters, facilitating more rapid WRKY rebinding and accelerated transcriptional reactivation upon re-stress. Together, these chromatin states illustrate how epigenetic context functions as a regulatory gate that shapes WRKY occupancy and context-dependent transcriptional specificity.

Epigenetic priming and stress memory can bias future accessibility and response kinetics, supporting the models based on prior stress exposure that establish a poised chromatin configuration that facilitates rapid reactivation of specific genomic loci upon stress [36]. Chromatin state constitutes a primary gate controlling WRKY access to W-box elements, operating upstream of DNA sequence recognition. Epigenetic features such as DNA methylation, histone modifications, and chromatin remodeling define whether W-boxes are inaccessible, actively engaged, or poised for rapid reactivation following stress. These distinct chromatin configurations are summarized schematically in Fig. 1. Together, chromatin and epigenetic signals act as an upstream filter that decides which W-boxes are available to WRKYs under specific physiological states.

## Complex-dependent specificity via protein-protein interactions

### WRKY-WRKY combinatorial regulation

WRKY TFs rarely operate as isolated DNA-binding proteins; instead, their regulatory polarity and promoter selection are often shaped by interaction-dependent complex assembly [14]. WRKY-WRKY associations enable combinatorial regulation within the family, through distinct homo and heteromeric assemblies that modulate promoter selectivity, stabilize DNA binding, and diversify transcriptional outcomes [48]. These interactions are relevant to stress phenotypes, consistent with complex formation operating as a regulatory filter that converts shared W-box recognition into context-dependent gene control [25]. In Arabidopsis, WRKY18, WRKY40, and WRKY60 form a well-studied regulatory module, with prior work demonstrating physical and functional interactions among these proteins [49]. Building on this framework, Liu et al. [29] showed that these WRKYs cooperatively repress the ABA-responsive genes ABI4 and ABI5 through W-box-containing promoter regions, with their regulatory effects varying by target promoter and WRKY combination. This example illustrates how WRKY–WRKY interactions can convert a shared DNA-binding capacity into promoter-specific transcriptional outputs rather than relying on W-box recognition alone.

### Cross-family transcription factor interactions

Cross-family interactions embed WRKYs in broader transcriptional modules that integrate developmental signals, metabolic programs, and stress responses. WRKY-MYB coordination has been linked to defense-related outputs and specialized metabolism, illustrating how partner identity can redirect WRKY function toward specific pathways [45]. More directly, VvWRKY8 physically interacts with VvMYB14 and represses stilbene synthase gene expression by limiting VvMYB14 promoter engagement, demonstrating that cross-family protein interactions can alter target selection without requiring changes in WRKY DNA-binding specificity [26].

### Accessory cofactors: VQ-motif proteins and OBE complexes

VQ-motif proteins are prominent WRKY partners that play an important role in WRKY modulation activity, connecting upstream phosphorylation events to transcriptional outcomes [27]. Structural and mechanistic work has clarified the VQ protein engagement with WRKY domains to change function, supporting a model in which cofactor availability becomes a determinant of specificity [13, 17, 52]. Mechanistic studies further indicate that VQ proteins engage WRKY domains in a subgroup-selective manner, supporting the view that cofactor recognition contributes to WRKY functional specificity rather than merely enhancing transcription factor activity.

A complementary mechanism is provided by OBERON (OBE) histone-binding proteins. In Arabidopsis, group IId WRKY transcription factors interact with OBE proteins to form WRKY–OBE complexes that repress stress-responsive gene expression under non-stress conditions. The WRKY coiled-coil region mediates OBE binding, while the OBE PHD finger contributes both histone association and WRKY interaction, linking transcription factor activity directly to chromatin-associated repression. Disruption of these complexes increases expression of stress-responsive genes and enhances drought tolerance, demonstrating that WRKY specificity can be shaped by cofactor-dependent recruitment into chromatin-linked regulatory modules [18]. Together, VQ proteins and OBE complexes show that accessory cofactors can determine how WRKY proteins with similar DNA-recognition capacity are deployed in distinct signaling and chromatin contexts, thereby refining promoter selection and transcriptional output.

## Post-translational regulation and proteostasis as rapid specificity switches

Post-translational modifications (PTMs) enable rapid and reversible regulation of WRKY activity and can reshape partner compatibility, transcriptional competence, and promoter engagement. WRKY33 represents one of the most compelling examples illustrating how WRKY transcription factor specificity emerges from a regulatory code rather than from DNA binding alone. Although WRKY33 recognizes canonical W-box elements that are widely distributed across the genome, its functional role in plant immunity is tightly constrained by signaling context, post-translational modification, and chromatin accessibility. WRKY33 is phosphorylated by the MAP kinases MPK3 and MPK6 during pathogen challenge, a modification that is essential for full transcriptional activation of defense-related genes and phytoalexin biosynthesis [30]. Importantly, this phosphorylation does not merely increase WRKY33 activity globally, but selectively enables binding and activation at pathogen-responsive loci. As detailed in Sect. 3, WRKY33 occupancy is highly inducible and is constrained by stress-dependent chromatin remodeling at immune-responsive loci [6]. In addition, WRKY33 interacts with VQ-motif proteins and other cofactors, further refining promoter selection and regulatory polarity. Together, these features illustrate how WRKY33 specificity arises from the integration of signaling input, chromatin accessibility, and cofactor interactions rather than from W-box recognition alone.

Mechanistic studies indicate that phosphorylation can bias promoter-level outcomes, and merely increasing overall activity, supporting PTMs as specificity modifiers instead of “on/off” switches [30]. Integrative pathway syntheses emphasize that MAPK cascades can elicit distinct outputs via differential substrate targeting and phosphorylation dynamics, offering a framework for WRKY diversification [31].

WRKY proteostasis controls protein abundance and dwell time. Ubiquitin-mediated degradation is linked to WRKY function in stress and developmental contexts, with WRKY53 as a classic example of stability control shaping transcriptional programs [32]. Redox signaling also reshapes transcription factor behavior under stress, especially in immune contexts where oxidative cues change the regulatory networks through reversible modifications and redox-dependent signaling states [41]. WRKY53 provides a contrasting example in which transcriptional specificity is governed primarily by proteostasis and hormonal context rather than by stress-induced chromatin remodeling. WRKY53 is a key regulator of leaf senescence, yet its activity cannot be explained solely by transcriptional induction or W-box recognition. Instead, WRKY53 function is tightly regulated at the protein level through ubiquitin-mediated degradation, which controls protein abundance and temporal activity windows. In addition to proteostasis, WRKY53 operates within a finely balanced hormonal environment, particularly involving salicylic acid and jasmonic acid signaling pathways. Shifts in hormone balance alter WRKY53 stability, interaction partners, and transcriptional output, enabling the same transcription factor to exert distinct regulatory effects depending on developmental stage and physiological state. These features explain how WRKY53 can act as a context-specific regulator of senescence-associated genes despite binding broadly conserved cis-elements. This case highlights proteostasis and signaling balance as dominant layers of the WRKY regulatory code and demonstrates that specificity can arise even in the absence of dramatic changes in DNA-binding preference.

## Signaling context and dynamic rewiring of WRKY outputs

WRKY specificity is conditioned by the signaling environment, particularly hormone balance and stress combinations. WRKY-centered networks participate in hormone crosstalk and defense signaling, where salicylic acid, ethylene, jasmonate, and ABA can remodel downstream regulatory logic and shift transcriptional priorities [35]. In field conditions, plants commonly face combined stresses, including abiotic and biotic stresses, that can generate non-additive transcriptional programs. Foundational analyses of abiotic/biotic stress combinations established the principle that integrated stresses induce a unique response requiring dedicated network control [42]. Recent studies have demonstrated that combined stresses trigger emergent signaling regimes that reconfigure transcriptional networks, providing a conceptual basis for WRKY rewiring under stress conditions [51].

Time introduces an additional regulatory dimension: early and late phases of stress impose distinct transcription factors and control requirements, and target relationships can change across temporal windows, indicating that stress-responsive transcription is temporally phased, suggesting that WRKY-dependent outputs are shaped by the timing of DNA binding relative to post-translational modification status, chromatin accessibility, and partner availability [23]. Furthermore, sequential stress exposure encodes historical effects through priming mechanisms, linking earlier signals to later altered responsiveness [36]. Thus, signaling context governs not only the presence of WRKY factors but also their regulatory capacity, which is mobilized across temporal and stress-dependent trajectories.

## The WRKY regulatory code as an integrative model

A unifying interpretation emerging from the evidence discussed above is that WRKY transcriptional specificity is not encoded by any single molecular determinant. Instead, it arises from the combined action of multiple regulatory layers that function sequentially and cooperatively. In this model, cis-regulatory motif grammar defines a permissive set of potential WRKY-responsive loci, but this sequence-level potential is filtered by chromatin accessibility and epigenetic state, which determine whether W-box-containing regions are physically available for binding. Among accessible loci, protein–protein interactions refine promoter choice and regulatory polarity, while post-translational modifications and proteostasis tune WRKY activity, stability, and signal responsiveness. Finally, hormonal, developmental, and stress-specific signaling contexts determine when these layers are engaged and how they are combined into a transcriptional outcome. We refer to this integrated, context-dependent decision system as the WRKY regulatory code [4, 6, 14, 24, 30, 51].

**Fig. 2:**
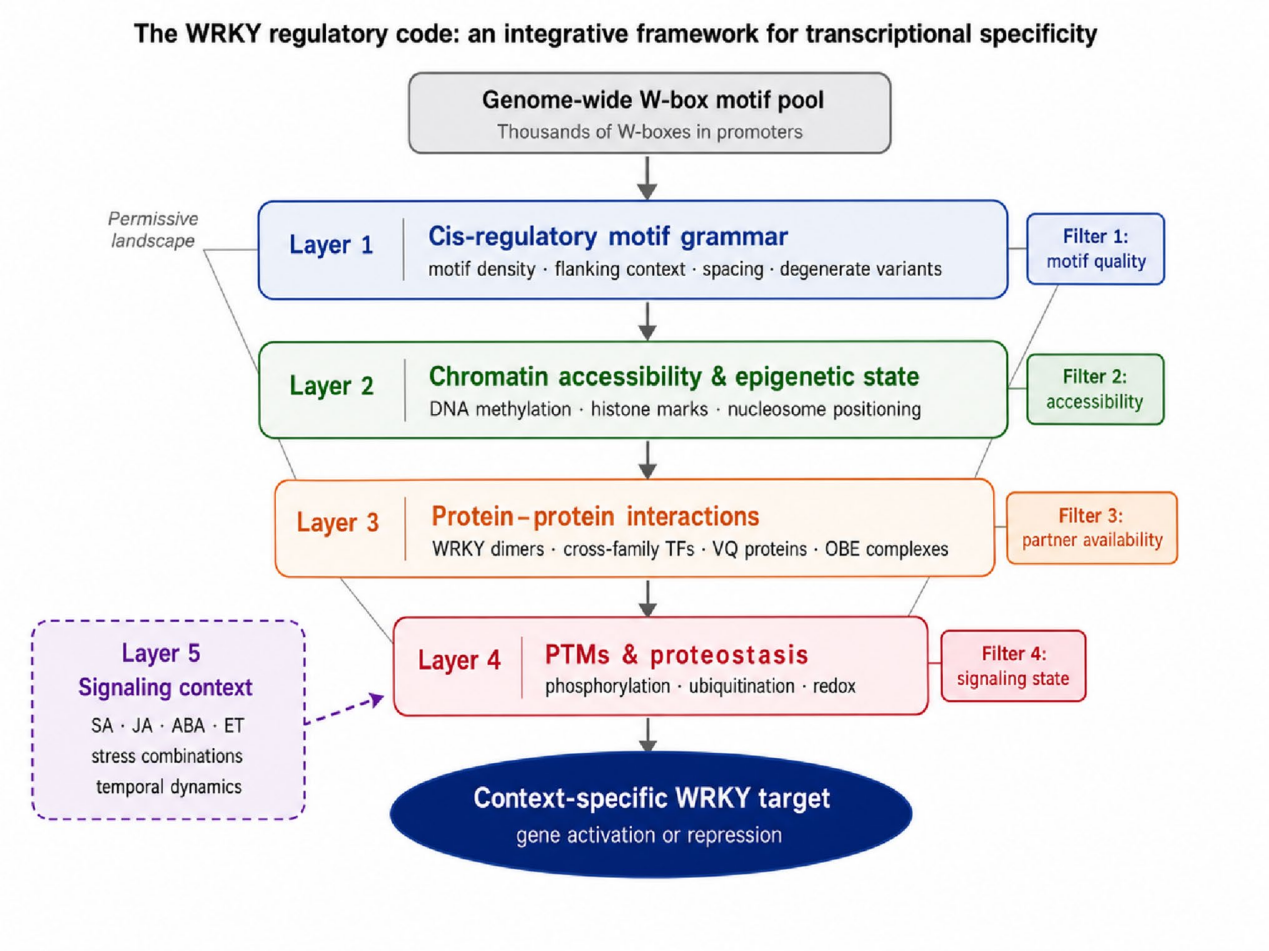
The WRKY regulatory code: an integrative framework for transcriptional specificity. WRKY target selection emerges from the combined action of five regulatory layers: cis-regulatory motif grammar, chromatin accessibility and epigenetic state, protein–protein interactions, post-translational modifications and proteostasis, and signaling context. Together, these filters determine which W-box-containing loci are occupied and whether the final transcriptional output is gene activation or repression

This framework resolves two recurring paradoxes in WRKY biology: first, why the widespread genomic abundance of W-box motifs poorly predicts actual WRKY occupancy or transcriptional regulation; and second, why the same WRKY protein can produce distinct outputs across tissues, stress conditions, and temporal phases [3, 35, 40]. Thus, the regulatory code concept does not imply a fixed universal formula, but rather a mechanistic framework in which different combinations of regulatory layers generate distinct WRKY outputs in specific biological contexts. An overview of these regulatory layers and their contributions to WRKY target selection is provided in Fig. 2; Table 1.

The operation of the WRKY regulatory code across physiological contexts is illustrated by representative examples summarized in Table 2. In biotic stress, WRKY33 demonstrates how signaling-dependent post-translational regulation, chromatin remodeling, and cofactor interactions converge to restrict WRKY activity to immune-responsive loci during pathogen challenge. In developmental and senescence-associated regulation, WRKY53 demonstrates that proteostasis and hormonal balance can serve as dominant specificity determinants rather than stress-induced chromatin changes. Under combined or sequential stresses, emergent signaling regimes may alter hormone balance, chromatin priming states, and cofactor availability, producing non-additive transcriptional programs that cannot be inferred from single-stress models. The WRKY–OBE complex further demonstrates how protein–protein interactions can shape regulatory specificity even under non-stress conditions. Collectively, these examples emphasize that the WRKY regulatory code does not operate through a single universal hierarchy; instead, different regulatory layers become dominant depending on physiological context [6, 18, 30, 32, 42, 51].

Importantly, the regulatory code model makes a stronger and more specific claim than the general statement that WRKY activity is regulated by multiple factors. Rather than simply listing independent modifiers, it specifies a sequential and combinatorial logic in which cis-regulatory grammar defines a permissive pool of candidate loci, chromatin accessibility filters this pool, and protein interactions, post-translational modifications, and signaling context then determine which accessible loci are productively engaged and whether the output is activation or repression. For example, the model predicts that experimentally opening chromatin at a methylated or nucleosome-occluded W-box should be necessary but not sufficient for WRKY-dependent activation unless the appropriate post-translational and cofactor conditions are also satisfied; that perturbing a single layer in isolation should shift, rather than abolish, target selection in a manner consistent with the remaining layers; and that condition-matched multi-omics measurements should predict WRKY occupancy and transcriptional output more accurately than W-box content alone. The framework is therefore falsifiable: it would be contradicted if WRKY target selection and regulatory polarity could be predicted from W-box sequence and abundance alone, or if the individual regulatory layers acted in a strictly additive, context-independent manner.

It is equally important to delineate the scope and current limitations of this framework. The mechanistic examples that anchor the model, most notably WRKY33, WRKY53, and the group IId WRKY–OBE complexes, are drawn from a small number of WRKY factors, largely studied in Arabidopsis. Whether the same layered logic and the relative dominance of particular layers generalizes across the full WRKY family and to crop species with larger gene families, distinct promoter architectures, and different chromatin landscapes remains to be established. The WRKY regulatory code should therefore be regarded as a unifying conceptual and predictive framework rather than a fixed universal formula, and its broad applicability across plant lineages will require systematic, multi-omics testing in additional WRKY subgroups and species. We anticipate that particular layers will prove more influential in specific taxa or stress regimes, and that the framework will be refined as comparative mechanistic data accumulate.

While the WRKY regulatory code provides a conceptual framework to explain how specificity emerges from multiple interacting regulatory layers, translating this framework into testable biology requires experimental approaches capable of resolving these layers simultaneously. Traditional single-factor analyses are insufficient to capture the combinatorial nature of WRKY regulation. Recent advances in high-throughput omics technologies now make it possible to empirically dissect how cis-regulatory features, chromatin accessibility, protein interactions, and post-translational modifications converge to shape WRKY-dependent transcriptional outputs. In this context, multi-omics strategies provide a critical bridge between conceptual models of WRKY specificity and mechanistic validation in vivo.

**Table 1:** Core regulatory layers of the WRKY regulatory code and their contributions to transcriptional specificity

Regulatory layer	Primary molecular features	Contribution to WRKY target specificity	Representative evidence
Cis-regulatory motif grammar	Canonical and degenerate W-boxes; motif density, clustering, spacing, and flanking sequence context	Establishes a permissive pool of potentially WRKY-responsive promoters and influences binding strength or combinatorial occupancy	Brand et al. [4], Ciolkowski [15], , Wu [47]
Chromatin accessibility and epigenetic state	DNA methylation, histone modifications, nucleosome positioning, chromatin remodeling, H2A.Z deposition	Determines whether W-box-containing loci are physically accessible for WRKY occupancy; creates inactive, active, or poised promoter states	Cai et al. [6], Charvin [8], Thiebaut [43]
Protein–protein interactions	WRKY–WRKY complexes, cross-family TF partnerships, VQ-motif proteins, OBE-associated complexes	Refines promoter selection, regulatory polarity, and chromatin-linked targeting through partner availability and complex composition	Chi et al. [14], Du [18], Jing [27], Liu [29]
Post-translational modifications and proteostasis	Phosphorylation, ubiquitination, redox-dependent modifications, protein turnover	Tunes WRKY activity, stability, signaling competence, and promoter engagement in a rapid and reversible manner	Ishihama et al. [24], Mao [30], Miao [32]
Signaling context and stress dynamics	Hormonal crosstalk, combined stresses, sequential stress exposure, temporal dynamics	Determines when regulatory layers are engaged and rewires WRKY outputs in a condition- and time-dependent manner	Phukan et al. [35], Ramakrishnan [36], Suzuki [42], Zandalinas [51]

**Table 2:** Representative examples illustrating how the WRKY regulatory code operates across stress and developmental contexts

Biological context	WRKY example	Dominant regulatory layers	Mechanistic implication for specificity
Biotic stress / plant immunity	WRKY33 in *Arabidopsis*	MAPK-dependent phosphorylation; chromatin remodeling at target loci; enrichment of active chromatin features; cofactor-dependent regulation	WRKY33 activity is restricted to immune-responsive loci through the combined effects of signaling input, chromatin accessibility, and partner interactions, demonstrating that W-box presence alone is insufficient for target activation.
Developmental / senescence regulation	WRKY53 in *Arabidopsis*	Protein stability controlled by ubiquitin-mediated turnover; modulation by salicylic acid–jasmonic acid balance	WRKY53 specificity is strongly shaped by proteostasis and hormonal context, allowing the same transcription factor to produce distinct outputs depending on developmental state and signaling environment.
Combined or sequential stress exposure	WRKY-centered stress networks	Hormone crosstalk; systemic stress signaling; stress-memory and priming-associated regulatory states	Complex or prior stress exposure can reconfigure signaling inputs and future responsiveness, generating transcriptional outputs that are not predictable from single-stress conditions alone.
Growth–stress balance / drought-associated repression	Group IId WRKYs–OBE complexes in *Arabidopsis*	WRKY–OBE protein complexes; OBE histone-binding activity; chromatin-associated repression of stress-responsive genes	Accessory cofactors can recruit WRKYs into chromatin-linked repressive complexes. Disruption of WRKY–OBE complexes derepresses stress-responsive genes and enhances drought tolerance, illustrating partner-dependent specificity beyond DNA recognition.

## Multi-omics approaches to dissect WRKY regulatory specificity

The increasing availability of high-resolution omics technologies has fundamentally reshaped how transcription factor function is studied in plants. For WRKY transcription factors, whose regulatory behavior cannot be explained by W-box recognition alone, multi-omics integration provides a powerful strategy to resolve the layered determinants of specificity proposed in the WRKY regulatory code framework. Rather than relying on single data types, recent studies increasingly combine transcriptomic, epigenomic, and proteomic datasets to identify when, where, and how WRKYs exert regulatory control [3, 46].

Transcriptomic profiling has been the primary approach for inferring WRKY function, revealing extensive stress-responsive expression patterns across plant species [35, 37]. However, bulk RNA-seq analyses often obscure WRKY specificity by averaging signals across heterogeneous cell populations and developmental stages. As a result, WRKY-dependent transcriptional outputs frequently appear pleiotropic or context inconsistent. Emerging single-cell RNA sequencing and spatial transcriptomics approaches provide resolution to this limitation by uncovering cell-type-restricted WRKY activity and stress-specific transcriptional modules that are not detectable in bulk datasets. These approaches reinforce the idea that WRKY specificity frequently arises from cellular context, cofactor availability, and chromatin state rather than from WRKY expression levels alone [3].

Epigenomic profiling adds a critical regulatory dimension to WRKY biology. Genome-wide chromatin accessibility assays such as ATAC-seq demonstrate that only a subset of genomic W-box elements are accessible under any given physiological condition, supporting chromatin gating as a dominant determinant of WRKY binding [7, 12, 43]. DNA methylation profiling further refines this view, as cytosine methylation at or near W-box motifs can directly interfere with WRKY-DNA interactions, rendering otherwise functional motifs inaccessible [8, 22]. ChIP-seq studies targeting WRKY transcription factors or histone modifications reveal that WRKY occupancy is highly dynamic and stress-dependent, correlating more closely with chromatin remodeling than with static promoter architecture [3].

Proteomic and phosphoproteomic approaches provide additional insight by capturing post-translational regulation and protein stability, which strongly influence WRKY activity. MAPK-mediated phosphorylation of WRKY proteins has been shown to be essential for activating defense-related transcriptional programs, linking upstream signaling pathways directly to WRKY-dependent gene regulation [24, 30]. Ubiquitin-mediated turnover and redox-dependent modifications further modulate WRKY protein abundance and functional state, allowing rapid, reversible tuning of transcriptional outputs without altering transcript levels [33, 41]. Together, these studies underscore the importance of integrating protein-level data with transcriptomic and epigenomic information.

**Fig. 3:**
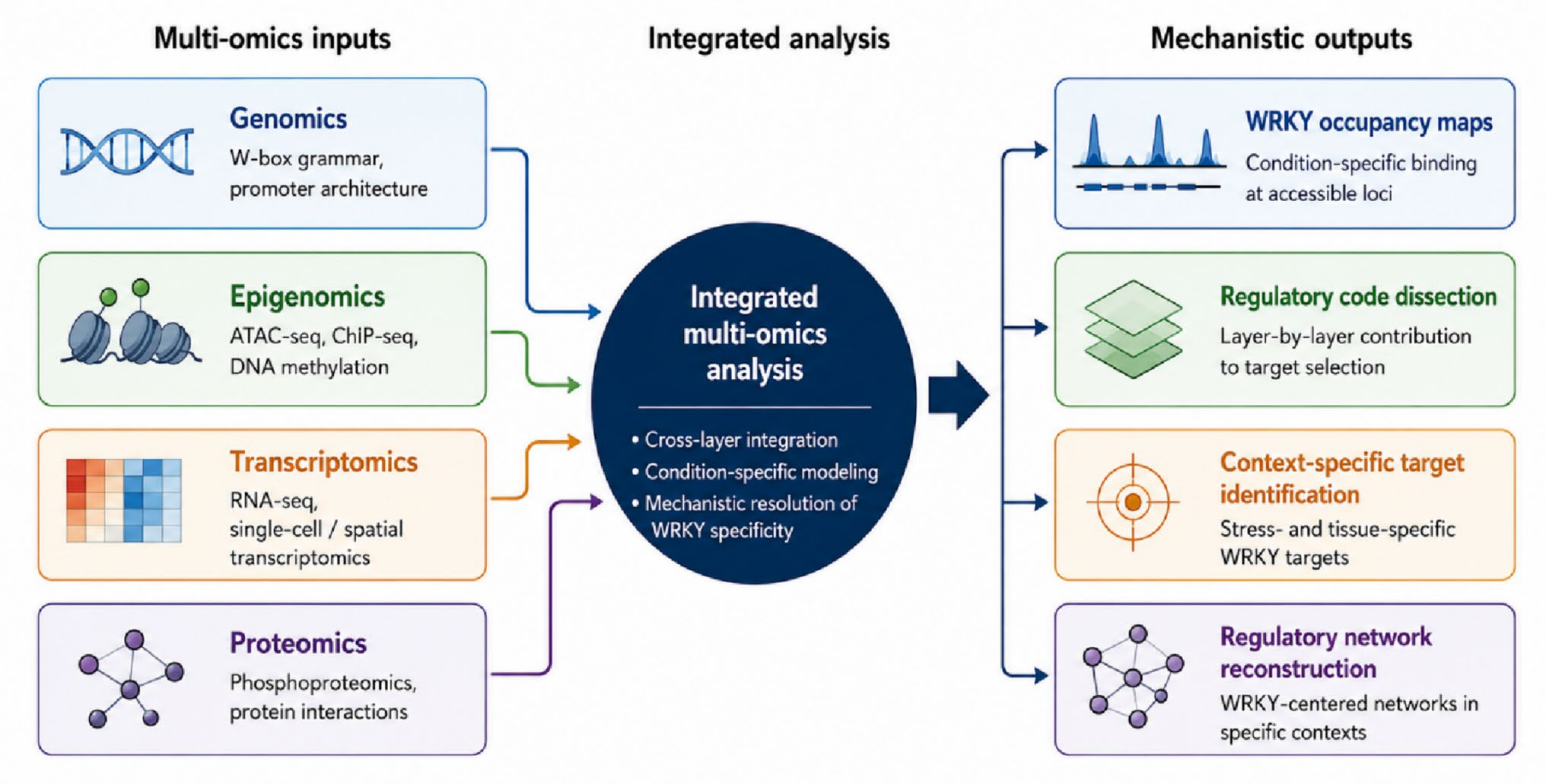
Multi-omics integration framework for decoding WRKY transcription factor specificity. Integration of genomic, epigenomic, transcriptomic, and proteomic datasets enables mechanistic dissection of the WRKY regulatory code. Cross-layer analysis helps define condition-specific WRKY occupancy, resolve layer-by-layer contributions to specificity, identify context-specific WRKY targets, and reconstruct WRKY-centered regulatory networks

Collectively, multi-omics approaches demonstrate that WRKY specificity is an emergent property of interacting regulatory layers rather than a consequence of any single molecular feature. Integrative analysis of these datasets provides a mechanistic foundation for testing the WRKY regulatory code hypothesis and enables condition-specific prediction of WRKY-dependent transcription across stresses, tissues, and developmental stages (Fig. 3).

Although multi-omics approaches provide unprecedented resolution of the molecular determinants underlying WRKY specificity, the complexity and dimensionality of these datasets present analytical challenges. The regulatory logic governing WRKY function is inherently combinatorial, nonlinear, and condition-dependent, making it difficult to infer predictive relationships using traditional analytical frameworks. Artificial intelligence and machine learning approaches offer a natural extension of multi-omics analysis by enabling integration of diverse regulatory features into unified predictive models. These approaches provide a pathway toward transforming descriptive insights into quantitative, testable predictions of WRKY regulatory behavior.

## Artificial intelligence and predictive modeling of WRKY regulatory networks

### Computational approaches in plant regulatory genomics

The combinatorial and nonlinear nature of WRKY transcriptional regulation makes it inherently suitable for machine learning and artificial intelligence based modeling. Traditional motif-centric approaches fail to predict WRKY target genes accurately because they do not account for chromatin accessibility, protein–protein interactions, post-translational modifications, or signaling context. In contrast, AI-driven models can learn complex relationships from high-dimensional multi-omics datasets, offering a path toward predictive understanding of WRKY-mediated gene regulation.

Existing regulatory-genomics models provide a foundation for this direction. PlantDeepSEA predicts the regulatory effects of non-coding variants on chromatin accessibility across six plant species and could help prioritize W-box-containing promoter variants relevant to WRKY regulation [53]. ChromBPNet, although not plant-specific, infers sequence determinants of chromatin accessibility, cooperative motif syntax, and transcription factor footprint-like patterns, offering a useful conceptual precedent for modeling WRKY cis-regulatory grammar [34]. In maize, GenomicLinks predicts three-dimensional chromatin interactions from sequence and identifies transcription factor motifs associated with loop specificity, suggesting a route to explore how WRKY-associated loci are embedded within higher-order regulatory architecture [39]. At present, these frameworks have not been applied directly to WRKY regulatory networks; their relevance should therefore be viewed as a near-term methodological opportunity rather than an established WRKY-specific application.

### Adapting AI frameworks to model WRKY specificity

Building on these computational advances, WRKY-specific predictive models could integrate feature classes that directly reflect the proposed regulatory code. These may include W-box sequence variants and promoter grammar [4, 15], chromatin accessibility and epigenetic state [8, 43], cofactor expression and protein interaction networks [14, 27], and post-translational modification status linked to stress signaling pathways [24, 31]. Interpretable supervised models could help evaluate the relative contribution of these regulatory layers, while deep learning architectures may capture higher-order sequence and chromatin patterns associated with condition-specific WRKY occupancy. Network-aware or graph-based approaches could further be explored to model how predicted WRKY regulatory relationships are rewired under combined or sequential stress conditions, a biological setting known to produce non-additive transcriptional responses [42, 51].

### A practical workflow for predictive WRKY target modeling

From an applied perspective, AI-driven prediction of WRKY regulatory outputs could provide a practical route for translating the WRKY regulatory code into testable, condition-specific hypotheses for crop improvement. Predictive models can identify promoters likely to respond to WRKY regulation only under specific environmental conditions, enabling precision promoter engineering and minimizing pleiotropic effects associated with constitutive WRKY manipulation [35, 46]. In this context, artificial intelligence provides a natural extension of the WRKY regulatory code framework, transforming qualitative regulatory principles into quantitative, testable, and predictive models.

**Fig. 4:**
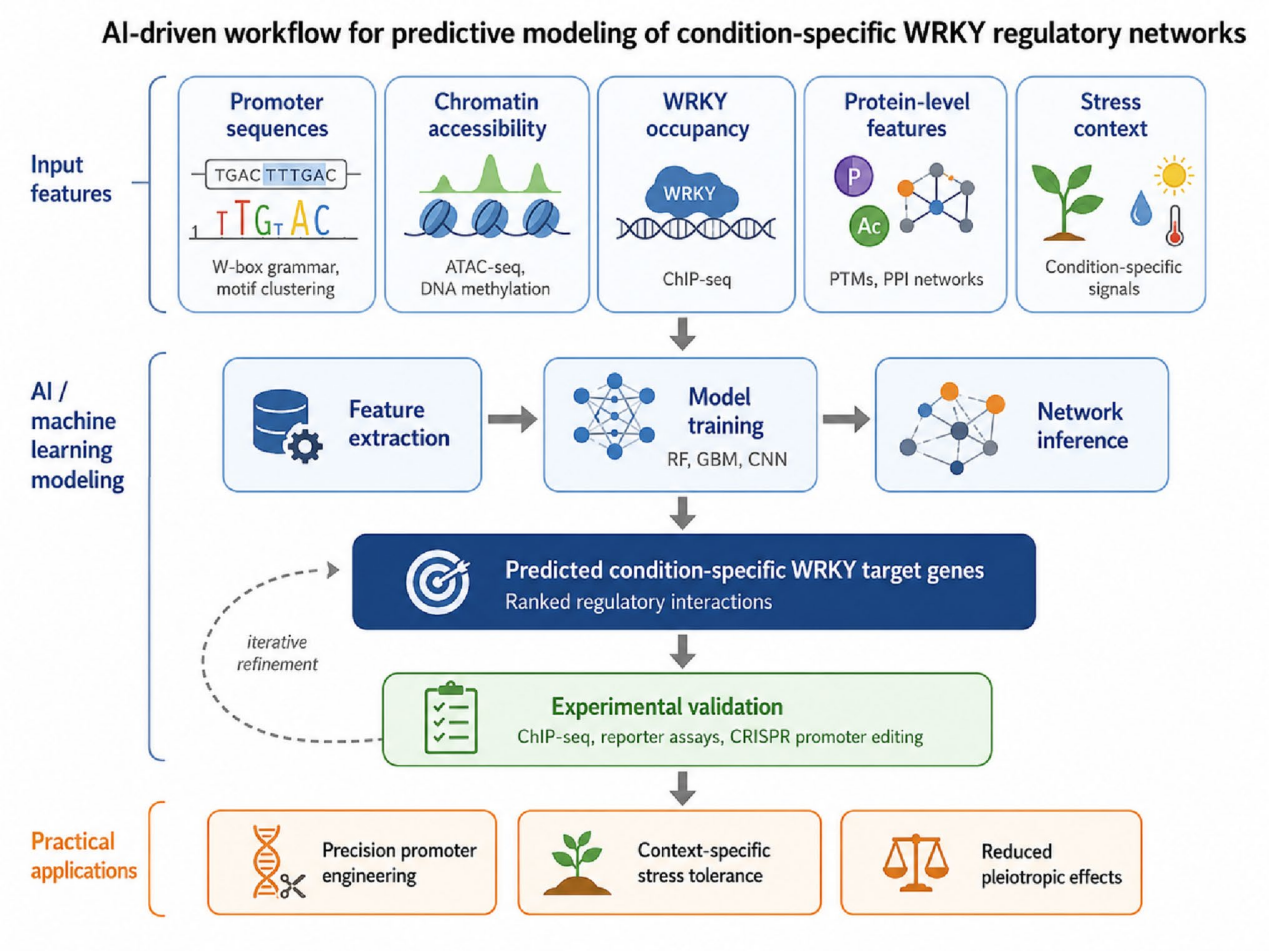
AI-driven workflow for predictive modeling of condition-specific WRKY regulatory networks. Promoter sequence features, chromatin accessibility, WRKY occupancy, protein-level regulatory information, and stress-context signals can be integrated into machine learning workflows for feature extraction, model training, and network inference. Predicted condition-specific WRKY target genes can then be prioritized for experimental validation and iterative model refinement, providing a route toward more predictive and testable models of WRKY regulatory specificity

A practical implementation of AI-driven prediction of WRKY regulatory targets would involve a stepwise workflow (Fig. 4): (i) identification of candidate promoters based on W-box grammar and motif clustering, (ii) filtering of accessible regulatory regions using chromatin accessibility data such as ATAC-seq, (iii) integration of WRKY occupancy, epigenetic, and proteomic features, including post-translational modification states, and (iv) training supervised machine learning models to predict condition-specific WRKY target genes. Predictions can then be experimentally validated and iteratively refined, enabling a closed-loop framework that couples computational inference with empirical testing. Such workflows move WRKY research from descriptive association toward predictive and programmable transcriptional control.

## Future directions and applications

An important implication of the WRKY regulatory code framework is that transcription factor specificity is likely to evolve more rapidly at regulatory layers than at the level of protein-coding sequences. WRKY DNA-binding domains are highly conserved across land plants, yet orthologous WRKY proteins frequently exhibit divergent regulatory roles across species and even within crop cultivars. This apparent paradox can be resolved by considering evolutionary changes in cis-regulatory grammar, chromatin landscapes, and interaction networks rather than alterations in DNA-binding specificity. Comparative genomics and epigenomics suggest that diversification of WRKY function across plant lineages is driven primarily by evolution of promoter architecture, chromatin accessibility patterns, and cofactor availability. These regulatory features are highly plastic and responsive to selection during adaptation and domestication, providing a mechanism for rapid functional divergence without compromising core DNA-binding capacity. Incorporating comparative and evolutionary perspectives into WRKY research will therefore be essential for understanding how regulatory codes are rewired across species and for transferring WRKY-based insights from model plants to crops.

A practical benefit of the regulatory code points to testable, multi-layer hypotheses and to translational strategies that are more precise than single-gene overexpression. Predictive models of WRKY targeting will likely require integration of cis features with chromatin accessibility (e.g., ATAC-seq), methylation states, and condition-matched occupancy datasets, because motif scanning is intrinsically underdetermined. A second priority is resolution in space and time: bulk tissue measurements obscure cell-type-specific WRKY logic, so single-cell and spatial genomics reveal hidden specificity drivers, particularly through cell-state-restricted chromatin accessibility and partner availability. For crop improvement, the framework suggests shifting from manipulating WRKY coding sequences toward editing regulatory layers. For example, promoter engineering and tuning of epigenetic states may reduce pleiotropy and improve context-specific stress resilience.

## Concluding perspective

The concept of a WRKY regulatory code reframes WRKY transcription factors not as stress-responsive switches, but as dynamic integrators of multiple regulatory signals. Future research that embraces this complexity rather than simplifying it will be better positioned to achieve predictive understanding and practical applications. By combining mechanistic insight with emerging technologies, the next phase of WRKY research has the potential to move from descriptive characterization toward predictive and programmable transcriptional control.

## Data Availability

No datasets were generated or analysed during the current study.

## References

[1] Bakshi M, Oelmüller R (2014) WRKY transcription factors: Jack of many trades in plants. Plant Signal Behav, 9(2), e27700 10.4161/psb.27700 PMC4091213 24492469

[2] Banerjee A, Roychoudhury A (2015) WRKY proteins: signaling and regulation of expression during abiotic stress responses. Sci World J 2015:807560 10.1155/2015/807560 PMC4387944 25879071

[3] Birkenbihl RP, Kracher B, Roccaro M, Somssich IE (2017) Induced genome-wide binding of three Arabidopsis WRKY transcription factors during early MAMP-triggered immunity. Plant Cell 29(1):20–38 28011690 10.1105/tpc.16.00681 PMC5304350

[4] Brand LH, Fischer NM, Harter K, Kohlbacher O, Wanke D (2013) Elucidating the evolutionary conserved DNA-binding specificities of WRKY transcription factors by molecular dynamics and in vitro binding assays. Nucleic Acids Res 41(21):9764–9778 23975197 10.1093/nar/gkt732 PMC3834811

[5] Buszewicz D, Archacki R, Palusiński A, Kotliński M, Fogtman A, Iwanicka-Nowicka R, Koblowska MK (2016) HD2C histone deacetylase and a SWI/SNF chromatin remodelling complex interact and both are involved in mediating the heat stress response in Arabidopsis. Plant Cell Environ 39(10):2108–2122 27083783 10.1111/pce.12756

[6] Cai H, Huang Y, Chen F, Liu L, Chai M, Zhang M, Yan M, Aslam M, He Q, Qin Y (2021) ERECTA signaling regulates plant immune responses via chromatin-mediated promotion of WRKY33 binding to target genes. New Phytol 230(2):737–756. 10.1111/nph.17200 33454980 10.1111/nph.17200

[7] Chang YN, Zhu C, Jiang J, Zhang H, Zhu JK, Duan CG (2020) Epigenetic regulation in plant abiotic stress responses. J Integr Plant Biol 62(5):563–580 31872527 10.1111/jipb.12901

[8] Charvin M, Halter T, Blanc-Mathieu R, Barraud P, Aumont-Nicaise M, Parcy F, Navarro L (2023) Single-cytosine methylation at W-boxes repels binding of WRKY transcription factors through steric hindrance. Plant Physiol 192(1):77–84 36782389 10.1093/plphys/kiad069 PMC10152670

[9] Chen H, Lai Z, Shi J, Xiao Y, Chen Z, Xu X (2010) Roles of Arabidopsis WRKY18, WRKY40 and WRKY60 transcription factors in plant responses to abscisic acid and abiotic stress. BMC Plant Biol 10:281 21167067 10.1186/1471-2229-10-281 PMC3023790

[10] Chen X, Lu L, Mayer KS, Scalf M, Qian S, Lomax A, Zhong X (2016) POWERDRESS interacts with HISTONE DEACETYLASE 9 to promote aging in Arabidopsis. eLife 5:e17214 27873573 10.7554/eLife.17214 PMC5119886

[11] Chen F, Hu Y, Vannozzi A, Wu K, Cai H, Qin Y, Zhang L (2017) The WRKY transcription factor family in model plants and crops. CRC Crit Rev Plant Sci 36(5–6):311–335

[12] Chen C, Ge Y, Lu L (2023) Opportunities and challenges in the application of single-cell and spatial transcriptomics in plants. Front Plant Sci 14:1185377. 10.3389/fpls.2023.1185377 37636094 10.3389/fpls.2023.1185377 PMC10453814

[13] Cheng Y, Zhou Y, Yang Y, Chi Y-J, Zhou J, Chen J-Y, Wang F, Fan B, Shi K, Zhou Y-H, Yu J-Q, Chen Z (2012) Structural and functional analysis of VQ motif-containing proteins in *Arabidopsis* as interacting proteins of WRKY transcription factors. Plant Physiol 159(2):810–825. 10.1104/pp.112.196816 22535423 10.1104/pp.112.196816 PMC3375943

[14] Chi Y, Yang Y, Zhou Y, Zhou J, Fan B, Yu JQ, Chen Z (2013) Protein–protein interactions in the regulation of WRKY transcription factors. Mol Plant 6(2):287–300 23455420 10.1093/mp/sst026

[15] Ciolkowski I, Wanke D, Birkenbihl RP, Somssich IE (2008) Studies on DNA-binding selectivity of WRKY transcription factors lend structural clues into WRKY-domain function. Plant Mol Biol 68(1):81–92 18523729 10.1007/s11103-008-9353-1 PMC2493524

[16] Dong J, Chen C, Chen Z (2003) Expression profiles of the Arabidopsis WRKY gene superfamily during plant defense response. Plant Mol Biol 51(1):21–37 12602888 10.1023/a:1020780022549

[17] Dong X, Yu L, Zhang Q, Yang J, Gong Z, Niu X, Li H, Zhang X, Liu M, Jin C, Hu Y (2024) Structural basis for the regulation of plant transcription factor WRKY33 by the VQ protein SIB1. Commun Biology 7:561. 10.1038/s42003-024-06258-7 10.1038/s42003-024-06258-7 PMC11088704 38734744

[18] Du P, Wang Q, Yuan D-Y, Chen S-S, Su Y-N, Li L, Chen S, He X-J (2023) WRKY transcription factors and OBERON histone-binding proteins form complexes to balance plant growth and stress tolerance. EMBO J 42(19):e113639. 10.15252/embj.2023113639 37565504 10.15252/embj.2023113639 PMC10548177

[19] Duan MR, Nan J, Liang YH, Mao P, Lu L, Li L, Su XD (2007) DNA binding mechanism revealed by high resolution crystal structure of Arabidopsis thaliana WRKY1 protein. Nucleic Acids Res 35(4):1145–1154 17264121 10.1093/nar/gkm001 PMC1851648

[20] Eulgem T, Somssich IE (2007) Networks of WRKY transcription factors in defense signaling. Curr Opin Plant Biol 10(4):366–371 17644023 10.1016/j.pbi.2007.04.020

[21] Eulgem T, Rushton PJ, Robatzek S, Somssich IE (2000) The WRKY superfamily of plant transcription factors. Trends Plant Sci 5(5):199–206 10785665 10.1016/s1360-1385(00)01600-9

[22] Gallego-Bartolomé J (2020) DNA methylation in plants: mechanisms and tools for targeted manipulation. New Phytol 227(1):38–44 32159848 10.1111/nph.16529

[23] Huang Y, Sun Z, Zhou X (2024) WRKY transcription factors in response to metal stress in plants: A review. Int J Mol Sci 25(20):10952 39456735 10.3390/ijms252010952 PMC11506853

[24] Ishihama N, Yamada R, Yoshioka M, Katou S, Yoshioka H (2011) Phosphorylation of the Nicotiana benthamiana WRKY8 transcription factor by MAPK functions in the defense response. Plant Cell 23(3):1153–1170 21386030 10.1105/tpc.110.081794 PMC3082260

[25] Jiang J, Ma S, Ye N, Jiang M, Cao J, Zhang J (2017) WRKY transcription factors in plant responses to stresses. J Integr Plant Biol 59(2):86–101 27995748 10.1111/jipb.12513

[26] Jiang J, Xi H, Dai Z, Lecourieux F, Yuan L, Liu X, Patra B, Wei Y, Li S, Wang L, Delrot S, Cheng L (2019) VvWRKY8 represses stilbene synthase genes through direct interaction with VvMYB14 to control resveratrol biosynthesis in grapevine. J Exp Bot 70(2):715–729. 10.1093/jxb/ery396 30445464 10.1093/jxb/ery401 PMC6322584

[27] Jing Y, Lin R (2015) The VQ motif-containing protein family of plant-specific transcriptional regulators. Plant Physiol 169(1):371–378 26220951 10.1104/pp.15.00788 PMC4577417

[28] Khoso MA, Hussain A, Ritonga FN, Ali Q, Channa MM, Alshegaihi RM, Manghwar H (2022) WRKY transcription factors (TFs): Molecular switches to regulate drought, temperature, and salinity stresses in plants. Front Plant Sci 13:1039329 36426143 10.3389/fpls.2022.1039329 PMC9679293

[29] Liu Z-Q, Yan L, Wu Z, Mei C, Lu K, Yu Y-T, Liang S, Zhang X-F, Wang X-F, Zhang D-P (2012) Cooperation of three WRKY-domain transcription factors WRKY18, WRKY40, and WRKY60 in repressing two ABA-responsive genes ABI4 and ABI5 in *Arabidopsis*. J Exp Bot 63(18):6371–6392. 10.1093/jxb/ers293 23095997 10.1093/jxb/ers293 PMC3504491

[30] Mao G, Meng X, Liu Y, Zheng Z, Chen Z, Zhang S (2011) Phosphorylation of a WRKY transcription factor by two pathogen-responsive MAPKs drives phytoalexin biosynthesis in Arabidopsis. Plant Cell 23(4):1639–1653 21498677 10.1105/tpc.111.084996 PMC3101563

[31] Meng X, Zhang S (2013) MAPK cascades in plant disease resistance signaling. Annu Rev Phytopathol 51:245–266 23663002 10.1146/annurev-phyto-082712-102314

[32] Miao Y, Zentgraf U (2007) The antagonist function of Arabidopsis WRKY53 and ESR/ESP in leaf senescence is modulated by the jasmonic and salicylic acid equilibrium. Plant Cell 19(3):819–830 17369373 10.1105/tpc.106.042705 PMC1867371

[33] Miao Y, Zentgraf U (2010) A HECT E3 ubiquitin ligase negatively regulates *Arabidopsis* leaf senescence through degradation of the transcription factor WRKY53. Plant J 63(2):179–188. 10.1111/j.1365-313X.2010.04233.x 20409006 10.1111/j.1365-313X.2010.04233.x

[34] Pampari A, Shcherbina A, Kvon EZ, Kosicki M, Nair S, Kundu S, Kathiria AS, Risca VI, Kuningas K, Alasoo K, Greenleaf WJ, Pennacchio LA, Kundaje AB (2025) ChromBPNet: Bias factorized, base-resolution deep learning models of chromatin accessibility reveal cis-regulatory sequence syntax, transcription factor footprints and regulatory variants. bioRxiv. 10.1101/2024.12.25.630221 39829783 10.1101/2024.12.25.630221 PMC11741299

[35] Phukan UJ, Jeena GS, Shukla RK (2016) WRKY transcription factors: molecular regulation and stress responses in plants. Front Plant Sci 7:760 27375634 10.3389/fpls.2016.00760 PMC4891567

[36] Ramakrishnan M, Zhang Z, Mullasseri S, Kalendar R, Ahmad Z, Sharma A, Wei Q (2022) Epigenetic stress memory: A new approach to study cold and heat stress responses in plants. Front Plant Sci 13:1075279 36570899 10.3389/fpls.2022.1075279 PMC9772030

[37] Rushton PJ, Somssich IE, Ringler P, Shen QJ (2010) WRKY transcription factors. Trends Plant Sci 15(5):247–258 20304701 10.1016/j.tplants.2010.02.006

[38] Rushton DL, Tripathi P, Rabara RC, Lin J, Ringler P, Boken AK, Rushton PJ (2012) WRKY transcription factors: key components in abscisic acid signalling. Plant Biotechnol J 10(1):2–11 21696534 10.1111/j.1467-7652.2011.00634.x

[39] Schlegel L, Bhardwaj R, Shahryary Y, Demirtürk D, Marand AP, Schmitz RJ, Johannes F (2024) GenomicLinks: Deep learning predictions of 3D chromatin interactions in the maize genome. NAR Genomics Bioinf 6(3):lqae123. 10.1093/nargab/lqae123 10.1093/nargab/lqae123 PMC11420838 39318505

[40] Schluttenhofer C, Yuan L (2015) Regulation of specialized metabolism by WRKY transcription factors. Plant Physiol 167(2):295–306 25501946 10.1104/pp.114.251769 PMC4326757

[41] Spoel SH, Loake GJ (2011) Redox-based protein modifications: the missing link in plant immune signalling. Curr Opin Plant Biol 14(4):358–364 21454121 10.1016/j.pbi.2011.03.007

[42] Suzuki N, Rivero RM, Shulaev V, Blumwald E, Mittler R (2014) Abiotic and biotic stress combinations. New Phytol 203(1):32–43 24720847 10.1111/nph.12797

[43] Thiebaut F, Hemerly AS, Ferreira PCG (2019) A role for epigenetic regulation in the adaptation and stress responses of non-model plants. Front Plant Sci 10:246 30881369 10.3389/fpls.2019.00246 PMC6405435

[44] Tripathi P, Rabara RC, Rushton PJ (2014) A systems biology perspective on the role of WRKY transcription factors in drought responses in plants. Planta 239(2):255–266 24146023 10.1007/s00425-013-1985-y

[45] Vannozzi A, Wong DCJ, Höll J, Hmmam I, Matus JT, Bogs J, Lucchin M (2018) Combinatorial regulation of stilbene synthase genes by WRKY and MYB transcription factors in grapevine (*Vitis vinifera* L). Plant Cell Physiol 59(5):1043–1059 29529275 10.1093/pcp/pcy045

[46] Wani SH, Anand S, Singh B, Bohra A, Joshi R (2021) WRKY transcription factors and plant defense responses: latest discoveries and future prospects. Plant Cell Rep 40(7):1071–1085 33860345 10.1007/s00299-021-02691-8

[47] Wu W, Zhu S, Xu L, Zhu L, Wang D, Liu Y, Chen J (2022) Genome-wide identification of the *Liriodendron chinense* WRKY gene family and its diverse roles in response to multiple abiotic stress. BMC Plant Biol 22:25 35012508 10.1186/s12870-021-03371-1 PMC8744262

[48] Xie Z, Zhang ZL, Zou X, Huang J, Ruas P, Thompson D, Shen QJ (2005) Annotations and functional analyses of the rice WRKY gene superfamily reveal positive and negative regulators of abscisic acid signaling in aleurone cells. Plant Physiol 137(1):176–189 15618416 10.1104/pp.104.054312 PMC548849

[49] Xu X, Chen C, Fan B, Chen Z (2006) Physical and functional interactions between pathogen-induced *Arabidopsis* WRKY18, WRKY40, and WRKY60 transcription factors. Plant Cell 18(5):1310–1326. 10.1105/tpc.105.037523 16603654 10.1105/tpc.105.037523 PMC1456877

[50] Yamasaki K, Kigawa T, Inoue M, Tateno M, Yamasaki T, Yabuki T, Yokoyama S (2005) Solution structure of an Arabidopsis WRKY DNA binding domain. Plant Cell 17(3):944–956 15705956 10.1105/tpc.104.026435 PMC1069710

[51] Zandalinas SI, Fichman Y, Devireddy AR, Sengupta S, Azad RK, Mittler R (2020) Systemic signaling during abiotic stress combination in plants. Proc Natl Acad Sci 117(24):13810–13820 32471943 10.1073/pnas.2005077117 PMC7306788

[52] Zhang L, Wang Y, Ni Z, Yu Y (2025) Functional and mechanistic insights into plant VQ proteins in abiotic and biotic stress responses. Plants 14(24):3855 41470737 10.3390/plants14243855 PMC12736750

[53] Zhao H, Tu Z, Liu Y, Zong Z, Li J, Liu H, Xiong F, Zhan J, Hu X, Xie W (2021) PlantDeepSEA, a deep learning-based web service to predict the regulatory effects of genomic variants in plants. Nucleic Acids Res 49(W1):W523–W529. 10.1093/nar/gkab383 34037796 10.1093/nar/gkab383 PMC8262748

